# Detecting post-surgical recurrence in a patient with Cushing’s disease

**DOI:** 10.1007/s11060-013-1343-8

**Published:** 2014-02-12

**Authors:** Kelley J. Moloney, Jennifer U. Mercado, William H. Ludlam, Marc R. Mayberg

**Affiliations:** 1 Seattle Pituitary Center, Swedish Neuroscience Institute, 550 17th Ave, Suite 500, Seattle, WA 98122 USA; 2 Novartis Pharmaceuticals Corporation, East Hanover, NJ USA

To the Editor,

Cushing’s disease (CD), a rare disorder of chronic hypercortisolism caused by an adrenocorticotropic (ACTH)-secreting pituitary adenoma, is associated with increased mortality and complications including central obesity, diabetes mellitus, osteoporosis, and cardiovascular disease [1]. Transsphenoidal adenomectomy is generally first-line treatment for CD but confirmation of surgical success and diagnosis of recurrence can prove challenging [1]. We endeavor to raise awareness of the complex, insidious nature of CD recurrence through this illustrative case report.

**Fig. 1:**
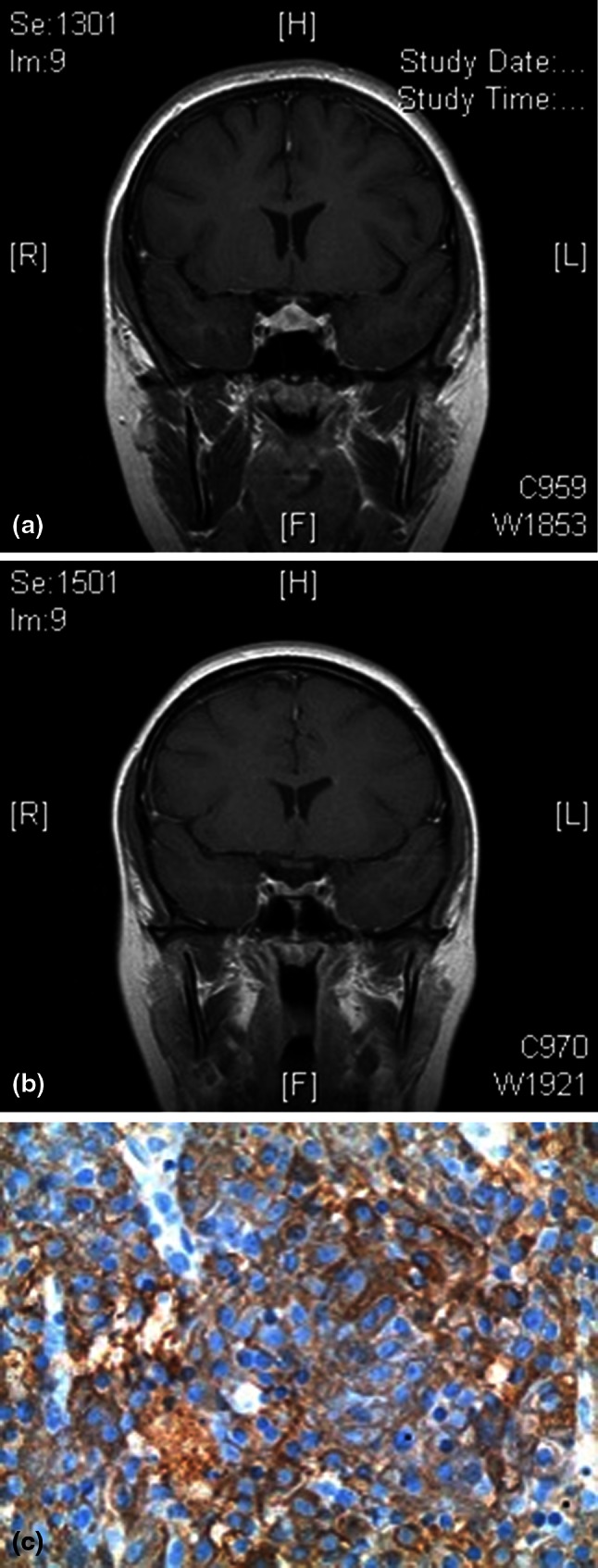
**a** Initial (2006) pre-operative T1 dynamic contrast-enhanced MRI (field strength 1.5 T) showing a 1.1-cm pituitary lesion. **b** Follow-up (2011) MRI with no lesion noted. **c** ACTH immunostain confirming recurrent CD

A 17-year-old female presented in 2006 with signs and symptoms of CD including facial rounding and rubor, hirsutism, alopecia, proximal muscle weakness, acne, stretch marks, weight gain, fatigue, insomnia, emotional fluctuations, headaches, and heartburn. She had elevated 24-hour urinary free cortisol [UFC; 126.5 μg/24 h (normal, <80)], and plasma ACTH [61 pg/mL (normal <48)]. Her dexamethasone suppression/corticotropin-releasing hormone stimulation test (Dex/CRH) was positive (serum cortisol 6.0 μg/dL 15 min after CRH) and magnetic resonance imaging (MRI) showed a 1.1-cm hypoenhancing pituitary lesion, confirming CD diagnosis (Fig. 1a).

After transsphenoidal surgery in December 2006, corticotroph adenoma was histologically confirmed. Consistent with CD remission, nadir serum cortisol measurement of 1.1 μg/dL (normal range 7.0–23.0) was recorded at 8 a.m. within 72 h post-surgery. Over the ensuing months, the patient reported decreased acne, heartburn, and proximal muscle weakness. Her face thinned and she lost 55 pounds.

Beginning 6 months after surgery, she reported that her previous symptoms began to return episodically several times per year. In February 2011, her symptoms (weight gain, acne, fatigue, and proximal muscle weakness) became unremitting. At this time, however, 24-h UFC and Dex/CRH tests were normal. She was noted to have elevated morning serum cortisol (peak, 31.5 μg/dL), but levels normalized after oral estrogen was discontinued. A follow-up MRI showed no pituitary lesion (Fig. 1b).

Despite consistently normal biochemical assessments (13 salivary cortisol tests, seven 24-h UFCs, three Dex/CRH tests, and one overnight Dex suppression test) over the 5-year period since pituitary surgery, the patient’s Dex/CRH levels ultimately became strongly positive (7.9 μg/dL 15 min after CRH; Dex level: 571 ng/dL) in late 2011. Based on the positive Dex/CRH result plus strong symptomatic and physical features of recurrent CD, the patient underwent repeat pituitary surgery. Corticotroph adenoma was again identified, removed, and confirmed by positive immunohistochemical staining for ACTH (Fig. 1c). Post-surgery, serum cortisol was measured every 6 h and, consistent with remission and possible complete removal of the adenoma, levels decreased to 0.9 μg/dL (normal 6.2–19.0) within 72 h. Her CD symptoms resolved and follow-up biochemistry was negative.

This case study illustrates the complexities of identifying patients with post-surgical recurrent CD. Within 4 years of apparent surgical remission, the patient experienced returning symptoms despite normal UFC and Dex/CRH tests. Ultimately, however, once she had biochemical evidence of CD recurrence (i.e., positive Dex/CRH despite continued normal UFC), she underwent repeat pituitary surgery and corticotroph adenoma was histologically confirmed. While subnormal immediate post-operative serum cortisol (as detected in this patient after her initial surgery) generally portends lasting remission, recurrence is still a possibility and occurs in approximately 6–12 % of such cases [2]. Similarly, normal UFC and/or midnight salivary cortisol levels are not reliable predictors of sustained remission per se. Careful follow up is required for all post-surgical CD patients and, if recurrence is suspected, biochemical test results should be interpreted in the context of clinical signs and symptoms.

CD has been associated with a 1.84- to 4.8-fold increase in mortality compared with the general population [3, 4]. However, many features of cortisol excess (e.g., obesity, depression, diabetes, hypertension, dyslipidemia) are common in the general population [1]. Given that 10-year post-surgical recurrence rates for CD may be as high as 56 % [5], postoperative CD patients should be meticulously monitored for persistence and recurrence of disease.

When this patient presented, no medical therapies were yet indicated for treatment of recurrent CD. Since that time, however, treatment options have expanded and clinicians have more choices for treating refractory cases. The US FDA has approved mifepristone (a glucocorticoid receptor antagonist) for treatment of hyperglycemia associated with Cushing’s syndrome [6], and pasireotide (a multireceptor-targeted somatostatin analog) for treatment of adults with CD who are not surgical candidates [7]. Choice of therapy depends on several factors including the patient’s overall surgical risk, severity of hypercortisolism, and patient preferences—particularly with respect to fertility. Regardless of treatment choice, special attention should be given to recurring symptoms as these may indicate early signs of disease even before biochemical evidence is present. Appropriate management is crucial, even when conflicting results make clinical decisions challenging.
